# Whole genome sequencing identified a 16 kilobase deletion on ECA13 associated with distichiasis in Friesian horses

**DOI:** 10.1186/s12864-020-07265-8

**Published:** 2020-11-30

**Authors:** E. A. Hisey, H. Hermans, Z. T. Lounsberry, F. Avila, R. A. Grahn, K. E. Knickelbein, S. A. Duward-Akhurst, M. E. McCue, T.S. Kalbfleisch, M. E. Lassaline, W. Back, R. R. Bellone

**Affiliations:** 1 Veterinary Genetics Laboratory, School of Veterinary Medicine, University of California-Davis, Davis, CA USA; 2 Department of Clinical Sciences, Utrecht University, Yalelaan 112-114, NL-3584 CM Utrecht, The Netherlands; 3 Veterinary Medical Teaching Hospital, University of California-Davis, Davis, CA USA; 4 Department of Veterinary Population Medicine, University of Minnesota, Saint Paul, MN USA; 5 Department of Veterinary Science, Gluck Equine Research Center, University of Kentucky, Lexington, KY USA; 6 Department of Clinical Sciences and Advanced Medicine, School of Veterinary Medicine, University of Pennsylvania, Philadelphia, PA USA; 7 Department of Surgery and Anaesthesia of Domestic Animals, Ghent University, Merelbeke, Belgium; 8 Department of Population Health and Reproduction, School of Veterinary Medicine, University of California-Davis, Davis, CA USA

**Keywords:** Genome wide association study (GWAS), Distichiasis, Meibomian gland, Haplotype, Whole genome sequencing (WGS), Functional annotation of animal genomes (FAANG), Histone marks, Eyelash

## Abstract

**Background:**

Distichiasis, an ocular disorder in which aberrant cilia (eyelashes) grow from the opening of the Meibomian glands of the eyelid, has been reported in Friesian horses. These misplaced cilia can cause discomfort, chronic keratitis, and corneal ulceration, potentially impacting vision due to corneal fibrosis, or, if secondary infection occurs, may lead to loss of the eye. Friesian horses represent the vast majority of reported cases of equine distichiasis, and as the breed is known to be affected with inherited monogenic disorders, this condition was hypothesized to be a simply inherited Mendelian trait.

**Results:**

A genome wide association study (GWAS) was performed using the Axiom 670 k Equine Genotyping array (MNEc670k) utilizing 14 cases and 38 controls phenotyped for distichiasis. An additive single locus mixed linear model (EMMAX) approach identified a 1.83 Mb locus on ECA5 and a 1.34 Mb locus on ECA13 that reached genome-wide significance (p_corrected_ = 0.016 and 0.032, respectively). Only the locus on ECA13 withstood replication testing (*p* = 1.6 × 10^− 5^, cases: *n* = 5 and controls: *n* = 37). A 371 kb run of homozygosity (ROH) on ECA13 was found in 13 of the 14 cases, providing evidence for a recessive mode of inheritance. Haplotype analysis (hapQTL) narrowed the region of association on ECA13 to 163 kb. Whole-genome sequencing data from 3 cases and 2 controls identified a 16 kb deletion within the ECA13 associated haplotype (ECA13:g.178714_195130del). Functional annotation data supports a tissue-specific regulatory role of this locus. This deletion was associated with distichiasis, as 18 of the 19 cases were homozygous (*p* = 4.8 × 10^− 13^). Genotyping the deletion in 955 horses from 54 different breeds identified the deletion in only 11 non-Friesians, all of which were carriers, suggesting that this could be causal for this Friesian disorder.

**Conclusions:**

This study identified a 16 kb deletion on ECA13 in an intergenic region that was associated with distichiasis in Friesian horses. Further functional analysis in relevant tissues from cases and controls will help to clarify the precise role of this deletion in normal and abnormal eyelash development and investigate the hypothesis of incomplete penetrance.

**Supplementary Information:**

The online version contains supplementary material available at 10.1186/s12864-020-07265-8.

## Background

Eyelashes serve to protect the eye from airborne particles and to prevent other debris from entering the eye. During embryonic development, the epidermal cells interact with the mesenchyme to form hair follicles, all of which are present at birth, with no additional follicles forming later in life [1, 2]. Like other body hairs, the growth of eyelashes follows a cyclical pattern. The growth cycle of the eyelash in humans is noted to be longer than body hairs, taking approximately 5 months to complete [3]. It has also been found that eyelashes have a shorter anagen phase and a longer telogen phase than other body hairs, contributing to the shorter length of the eyelashes [3].

Meibomian glands are holocrine glands present along the eyelid margin and are closely associated with the lash follicle, though eyelashes typically exit the skin anterior to the Meibomian gland orifice [4]. Meibomian glands are considered modified sebaceous glands because of the unique combination of lipids they secrete to assist in the prevention of evaporation of the tear film [5, 6].

Distichiasis is a condition in which eyelashes exit through the Meibomian gland orifice [5, 7]. In cases of distichiasis, it is hypothesized that the Meibomian glands have regained an ancestral hair-bearing function, and thus, a lash grows from the opening [5, 8]. Another hypothesis is that a primary epithelial germ cell fails to differentiate into a sebaceous gland and instead becomes a complete pilosebaceous unit, which is associated with a hair [9]. Meibomian glands affected with distichiae are structurally abnormal based on meibography [6]. While distichiae can be shorter, thinner and less pigmented than normal eyelashes [8], they can also be thick and stiff and thus capable of causing tearing, corneal irritation, keratitis, and corneal erosions or ulcers, which can impact ocular comfort and vision, and may lead to secondary infection [5, 10–12].

Two novel dominant mutations in *forkhead box protein C2* (*FOXC2*) have been associated with distichiasis in humans [13, 14]. FOXC2 is a transcription factor that plays a major role during embryogenesis [14] although the precise regulatory function of this gene is not well understood. Both mutations are nonsense mutations, truncating the protein and impairing DNA-binding, which in turn prevents it from acting as a transcription factor. The complete list of genes regulated by FOXC2 is not known and these mutations only explain the disease in two families suggesting that other unexplained genetic mechanisms for distichiasis in humans may exist. Congenital distichiasis is also commonly seen in dogs. The mode of inheritance in this species is reported to be dominant with incomplete penetrance [15]. Despite its occurrence in multiple dog breeds, a genetic mechanism has not been identified for this disorder.

**Fig. 1:**
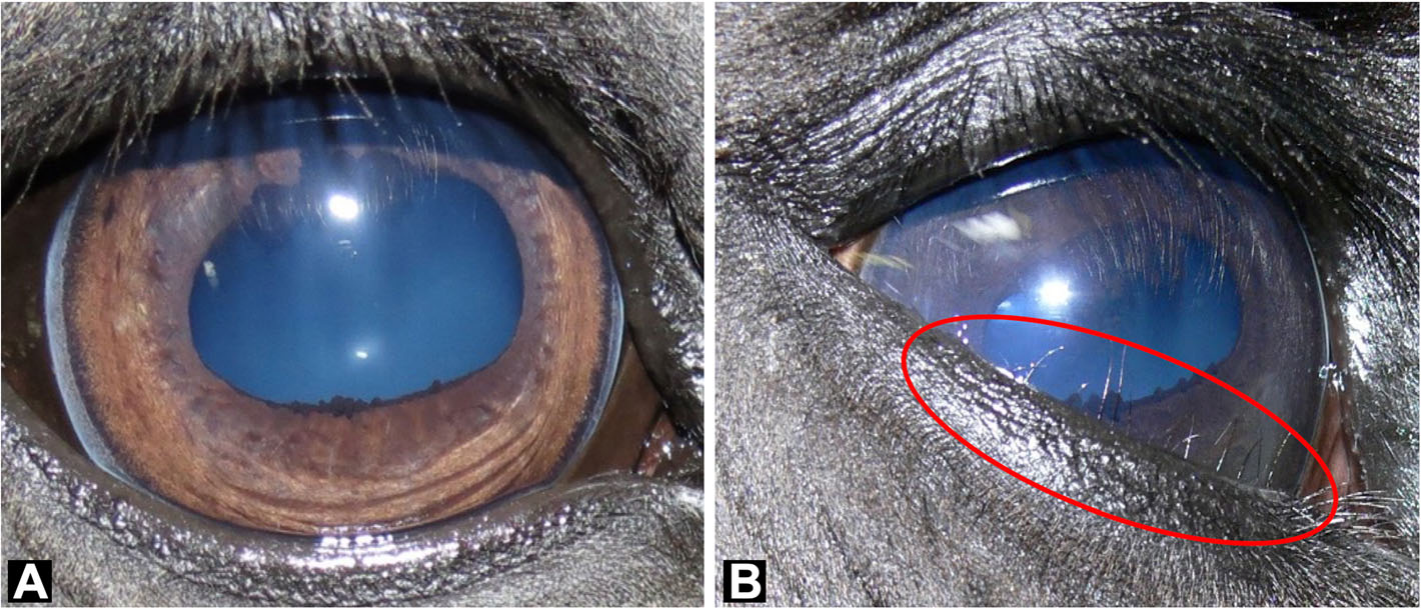
Distichiasis in a Friesian horse. **a** Normal eyelashes developing from the outer surface of the lid. **b** Aberrant eyelashes growing from the Meibomian gland orifices of the inferior eyelid margin. Note the eyelashes growing such that they are in direct contact with the cornea

In the two published reports of distichiasis in horses, 19 of the 20 cases were of the Friesian breed (Fig. 1) [11, 12]. Utter and Wotman [11] describe distichiasis causing recurrent corneal ulceration in two Friesian horses located in the United States. In a retrospective analysis, Hermans and Ensink [12] reported a high rate of recurrence despite treatment, particularly when individuals had five or more aberrant lashes, and that all cases presented with irritation or ulceration [12]. The number of Friesian cases presented in these two reports suggests a genetic basis for distichiasis in this breed.

Friesians have been shown to have a high degree of inbreeding [16]. Inbreeding is thought to be responsible for a number of suspected genetic disorders identified in this breed, including hydrocephalus, dwarfism, bilateral corneal stromal loss (BCSL), megaesophagus, retained placenta, aortic rupture, and chronic progressive lymphedema [17–24]. Recessive causal mutations have been identified for two of those disorders: hydrocephalus (*B3GALNT2*c.1423C > T) and dwarfism (*B4GALT7*c.50G > A) [17, 18]. As Friesians have been documented to have recessive Mendelian disorders, have a high level of inbreeding, and the incidence of distichiasis seems higher than in other breeds, it was hypothesized that in Friesian horses distichiasis is a recessively inherited disorder. To investigate this hypothesis, a genome wide association study (GWAS) was performed to identify candidate loci followed by whole genome sequencing (WGS) and functional analyses to identify a causal variant for distichiasis in Friesian horses.

## Results

### Pedigree analysis

**Fig. 2:**
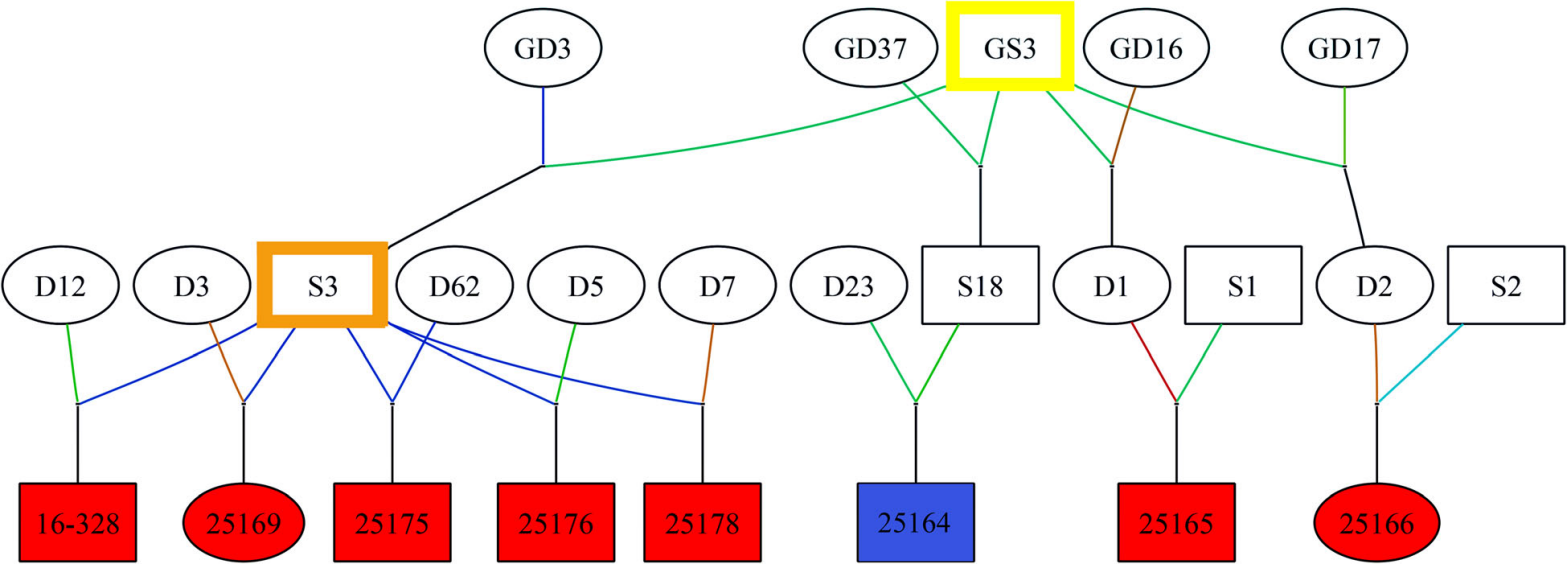
Distichiasis Pedigree Investigation. Represented is the most informative portion of the pedigree. Specifically, pedigree analysis showed that five of 21 affected horses shared a common ancestor within a single generation (S3, indicated with an orange border), which was not related to any of the controls. Two additional affected horses could be traced to the sire of S3 (GS3, indicated with a yellow border). Taken together, these findings support a genetic basis for this disease. Cases are denoted with red shading. Disease states of ancestors are unknown and denoted with white shading. One unaffected individual is denoted with blue shading

A pedigree analysis identified that 30% of cases traced back to a single common ancestor (GS3, Fig. 2) within two generations, providing support of a genetic predisposition for distichiasis. Specifically, five of the 21 cases were identified as half-siblings (offspring of S3, Fig. 2) and two additional cases traced back to his sire (GS3). The total inbreeding coefficients were calculated and compared between cases (Mdn = 0.144) and controls (Mdn = 0.147), with no statistically significant difference detected (*p* = 0.31 and U = 425). However, the inbreeding coefficient for all horses in this analysis was high at 14.1%.

### Genome wide association study

**Fig. 3:**
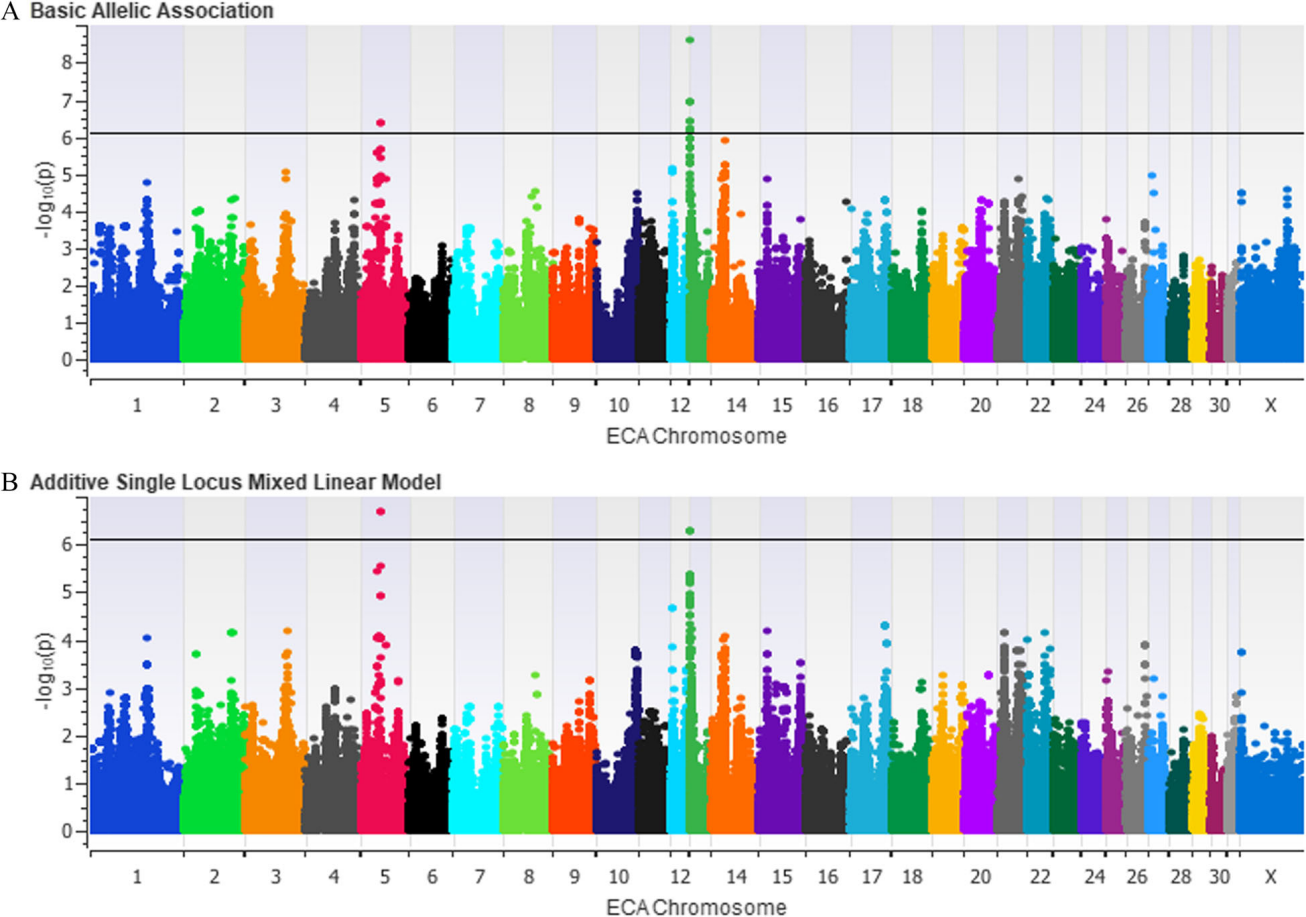
Manhattan Plots from GWAS Analyses. Plots are organized by chromosome (denoted by different colors). The modified Bonferroni correction threshold is indicated by the black line (−log_10_(p) = 6.08). **a** –log_10_(*p*-values) from a chi-squared analysis for basic allelic association (λ = 1.5) (**b**) –log_10_(p-values) from a single locus mixed linear model (EMMAX) analysis under an additive model (λ = 1.0). **b** Two loci reach genome wide significance, on ECA5 and ECA13 after correcting for genomic inflation (p_corrected_ = 0.016 and 0.032, respectively)

A chi-squared basic allelic association identified a 1.83 Mb locus on ECA5 and a 1.34 Mb locus on ECA13 that reached genome-wide significance (Fig. 3a). Two significant SNPs (p_corrected_ = 0.025) were found5s on the ECA5 locus, while the locus on ECA13 contained 16 significant SNPs (p_corrected_ < 0.042). However, the genomic inflation in this analysis was high (λ = 1.50). To correct for the unequal relatedness in the GWAS cohorts, an EMMAX analysis was performed. Under an additive model, the loci on ECA5 and ECA13 were further supported (p_corrected_ = 0.016 and p_corrected_ = 0.031, respectively; λ = 1.03, Fig. 3b).

Haplotype analysis of the ECA13 associated locus identified a 371 kb run of homozygosity (ROH) in 13 out of 14 cases, which was significantly associated with disease (chi-squared *p* = 3.9 × 10^− 9^, Table 1). This haplotype contained three genes: *FAM20C*, *PRKAR1B,* and *PDGFA*.

**Table 1:** Identification of Distichiasis Associated ROH

	n	Homozygous case major haplotype	Heterozygous	Homozygous case minor haplotype
Cases	14	13	1	0
Controls	38	4	17	17

A significant ROH was identified in the ECA13 associated locus (chi-squared test for independence, *p* = 3.9 × 10^− 9^)

### hapQTL

**Fig. 4:**
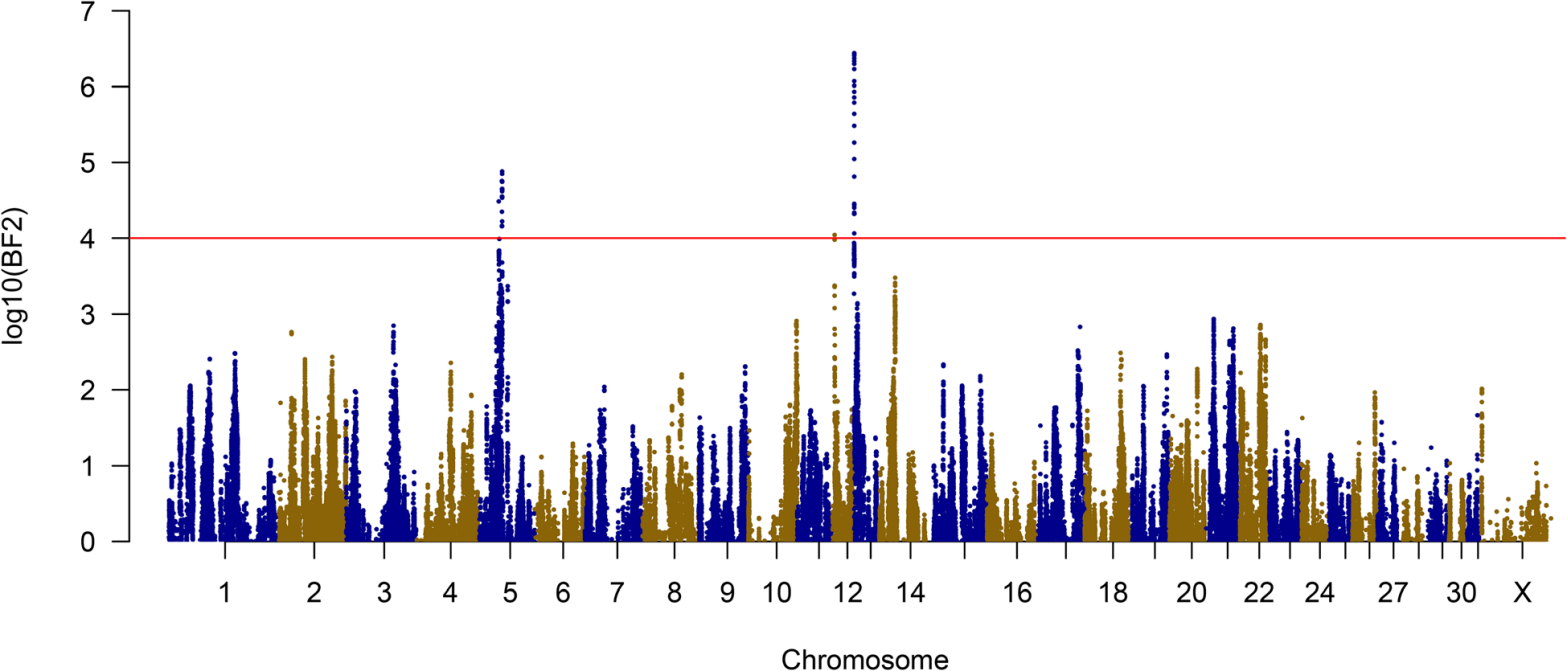
Haplotype Analysis. BF2 values from a genome-wide haplotype-based analysis using hapQTL. A significance threshold of log_10_(BF2) > 4 was utilized [25]. Haplotypes on ECA5, ECA12 and ECA13 were found to be significant, further supporting and narrowing the regions of association on ECA5 and ECA13 from the initial GWAS

Three significant ancestral haplotypes were also identified based on their Bayes Factor (BF) values of less than 0.0001 [25]: the same loci on ECA5 and ECA13, plus a locus on ECA12 (Fig. 4). This narrowed the candidate region on ECA5 to 235 kb and to 163 kb on ECA13. The locus on ECA12 contains one small haplotype spanning 375 bp, which encompassed only two SNPs.

### Replicating GWAS associations

To confirm genotyping results and replicate associations, SNPs that reached genome-wide significance in the GWAS (27 SNPs from ECA5 and ECA13) or haplotype analyses (2 SNPs from ECA12) were genotyped in all horses from the GWAS plus a replication sample set including five additional cases and 37 additional controls. In the replication sample set, the loci on ECA5 and ECA12 no longer reached significance (*p* > 0.40, Table 2). However, the ECA13 locus was further supported as it continued to reach genome wide significance in the replication sample set (*p* = 1.6 × 10^− 5^ and combined data set: *p* = 5.1 × 10^− 14^, Table 2).

**Table 2:** GWAS Replication Testing

Name	Original sample ***p***-value	Replication sample p-value	Combined p-value
ECA13:g.340918C > T	2.26 × 10^−8^	1.61 × 10^−5^	5.71 × 10^− 14^
ECA13:g.343850A > C	2.26 × 10^− 8^	1.61 × 10^− 5^	5.71 × 10^− 14^
ECA13:g.330462G > A	2.73 × 10^− 7^	2.34 × 10^− 5^	8.58 × 10^− 13^
ECA13:g.425443C > T	4.16 × 10^− 7^	1.45 × 10^− 4^	1.64 × 10^− 11^
ECA13:g.230097C > T	2.27 × 10^− 7^	2.30 × 10^− 4^	2.31 × 10^− 11^
ECA13:g.134040A > G	8.15 × 10^− 8^	3.07 × 10^− 4^	2.31 × 10^− 11^
ECA13:g.142416C > T	2.27 × 10^− 7^	3.07 × 10^− 4^	2.73 × 10^− 11^
ECA13:g.153435C > T	2.27 × 10^− 7^	3.07 × 10^− 4^	2.73 × 10^− 11^
ECA13:g.195278 T > C	2.27 × 10^− 7^	3.07 × 10^− 4^	2.73 × 10^− 11^
ECA13:g.198581G > C	2.27 × 10^− 7^	3.07 × 10^− 4^	2.73 × 10^− 11^
ECA13:g.200759 T > C	2.27 × 10^− 7^	3.07 × 10^− 4^	2.73 × 10^− 11^
ECA13:g.201534G > A	2.27 × 10^− 7^	3.07 × 10^− 4^	2.73 × 10^− 11^
ECA13:g.206996G > A	2.27 × 10^− 7^	4.06 × 10^− 4^	3.75 × 10^− 11^
ECA13:g.121325G > A	2.73 × 10^− 7^	1.07 × 10^− 3^	1.12 × 10^− 11^
ECA13:g.122064G > A	2.73 × 10^− 7^	1.07 × 10^− 3^	1.12 × 10^− 11^
ECA13:g.122243C > T	2.73 × 10^− 7^	1.07 × 10^− 3^	1.12 × 10^− 11^
ECA13:g.316346C > T	2.73 × 10^− 7^	1.07 × 10^− 3^	1.12 × 10^− 11^
ECA13:g.13611A > G	7.73 × 10^− 7^	1.10 × 10^− 3^	3.94 × 10^− 10^
ECA13:g.42897A > G	2.73 × 10^− 7^	1.10 × 10^− 3^	1.45 × 10^− 10^
ECA13:g.32112G > T	8.48 × 10^− 7^	1.39 × 10^− 3^	1.36 × 10^− 9^
ECA13:g.384663 T > C	5.94 × 10^− 6^	2.00 × 10^− 3^	5.77 × 10^− 9^
ECA13:g.15928A > G	8.15 × 10^− 8^	0.0288	2.04 × 10^− 9^
ECA5:g.39962594G > A	4.57 × 10^− 6^	0.404	4.65 × 10^− 9^
ECA5:g.39863319A > G^a^	4.57 × 10^− 6^	0.478	1.28 × 10^− 5^
ECA12:g.3271275C > A	9.10 × 10^− 6^	0.716	6.75 × 10^− 4^
ECA12:g.3270900 T > C	1.57 × 10^− 5^	1	4.65 × 10^− 4^
ECA13:g.186975G > A^b^	1	1	1
ECA13:g.189306C > T^b^	1	1	1
ECA13:g.190216 T > C^b^	1	1	1

^a^ This SNP was assessed through a PCR-RFLP analysis

^b^As these SNPs fall within the 16 kb deletion, they only yielded results in one case (see Fig. 5)

Genotyping the associated SNPs from the GWAS analyses was completed through a MassARRAY assay or a PCR-RFLP assay. To test for significance, a Fisher’s exact test for basic allelic associations was performed. Data from the original GWAS sample set (*n* = 14 case and *n* = 38 controls), the replication sample set (*n* = 5 cases and *n* = 37 controls), and the combined data set are presented.

### Whole genome sequencing

While *FOXC2* (ECA3) did not fall within a region of association in this GWAS, variants in this gene have been associated with distichiasis in humans [13, 14], thus it was investigated as a candidate gene for equine distichiasis. Analysis of WGS data from the coding region of this gene identified four variants (three intronic and one synonymous variant: ECA3:g.34103404C > T; rs1145785150), but none of these were concordant with phenotype in the three cases sequenced and therefore they were not investigated further.

Replication testing supported further investigation of the ECA13 locus, coding variants in the WGS data for the three ECA13 positional candidate genes, *FAM20C*, *PRKAR1B* and *PDGFA*, were investigated. No variants concordant with phenotype were identified in the three cases and two controls sequenced. However, 32 variants from the associated haplotype were prioritized for further analysis. These variants were either perfectly concordant with the phenotype in the WGS sample set (*n* = 3 cases and *n* = 2 controls) or were predicted by SnpEff to impact protein function (Table S1). These 32 variants were genotyped via Agena MassARRAY spectrometry in the cohort of Friesian horses. Five of these variants, which were predicted to be synonymous or modifier (intronic) variants, failed to genotype. Twenty variants failed to pass quality control (minor allele frequency < 0.05), indicating that these were not polymorphic in our sample set and thus were not segregating with disease status. The remaining seven variants were evaluated further (Table 3).

**Table 3:** Association Testing of Prioritized Variants Identified by Whole Genome Sequencing from the ECA13 Locus

Name	p-value
ECA13:g.178,714–195,130del^a^	1.90 × 10^− 15^
ECA13:g.117852G > A	2.31 × 10^− 11^
ECA13:g.127995G > A	2.31 × 10^− 11^
ECA13:g.134862C > G	2.31 × 10^− 11^
ECA13:g.138340G > A	2.74 × 10^−11^
ECA13:g.125711 T > C	1.12 × 10^−10^
ECA13:g.158596G > A	1.45 × 10^−8^
ECA13:g.710940 T > C	1

^a^This variant was genotyped by an allele specific PCR assay

Results of Fisher’s exact tests for basic allelic associations are presented.

**Fig. 5:**
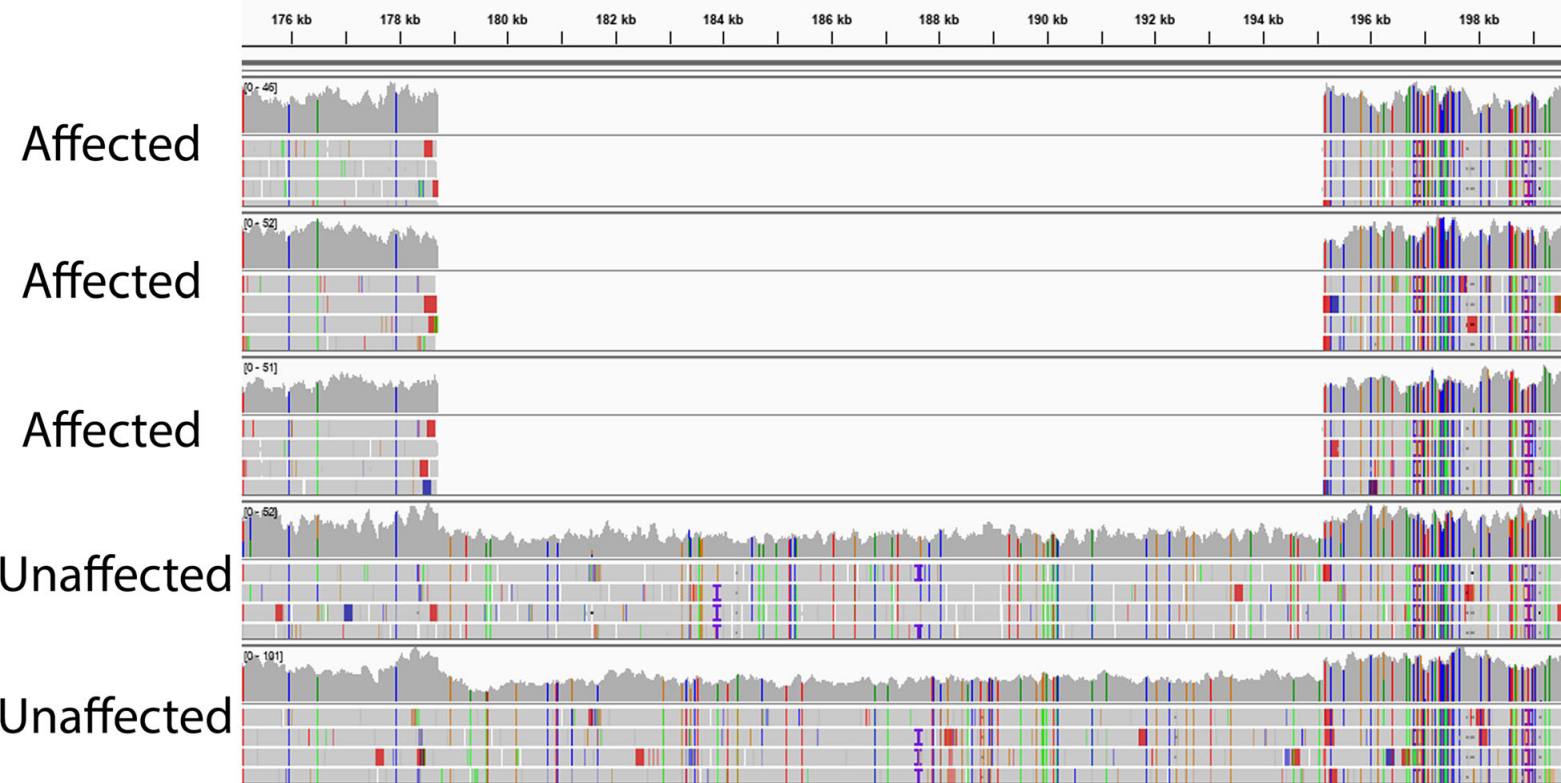
Visualization of Whole Genome Sequencing Data Identifies 16.42 kb Deletion. BAM files were visually inspected using the Integrative Genomics Viewer (IGV). No reads were detected in three cases within a 16.42 kb region (ECA13:g.178,714–195,130del), but were found in the controls at approximately half the coverage of the flanking sequence

Visual inspection of the WGS data from the associated ECA13 locus identified a deletion in this region. Specifically, the binary alignment files (BAM) were inspected using the Integrative Genomics Viewer (IGV). No coverage in a 16.42 kb region in this locus was detected in the cases; coverage was consistent across the two controls in this region with roughly half the number of reads as the flanking sequences, suggesting the two controls were heterozygous for this deletion (Fig. 5). This variant was later confirmed as the only structural variant in the ECA13 associated haplotype using LUMPY.

Sanger sequencing of the deletion in two cases and two controls identified the boundaries to occur at ECA13:g.178714_195130del. Further genotyping of the deletion using an allele specific PCR assay in our entire phenotyped sample (*n* = 94) determined that the deletion was significantly associated with phenotype as all but one case was homozygous for this structural variant (chi-squared *p* = 4.8 × 10^− 13^, Table 4). However, seven out of 75 controls were also homozygous for this variant. A random sample set of Friesian horses that were not phenotyped (*n* = 201, banked in the Bellone laboratory) were evaluated and the allele frequency was estimated to be 0.32 (Table 4). Due to the moderate allele frequency, the frequency for the two other reported recessive mutations in the Friesian breed was also assessed in a non-phenotyped subset of the sample (*n* = 73). Based on these data, the estimated allele frequencies were determined to be 0.075 for hydrocephalus and 0.062 for dwarfism. None of the other prioritized WGS variants in the associated locus on ECA13 were found to be as concordant with phenotype as the 16 kb deletion (Table 3).

**Table 4:** Validation of ECA13 16 kb Deletion in Sample of Friesian Horses

	n	Del/Del	Ref/Del	Ref/Ref
Cases	19	18	1	0
Controls	75	7	30	38
Unknown phenotype	201	21	88	92

Del refers to the ECA13 16 kb deletion and Ref signifies detection of the reference allele.

Genotyping for the deletion was completed through an allele specific PCR assay in the phenotyped cohort (*n* = 94) and in an additional sample set of horses that were not phenotyped for disease, which estimated the allele frequency to be 32.34%.

To determine if this deletion occurs in other breeds, 955 horse samples from 54 breeds were genotyped. These included 279 samples banked in the Bellone laboratory (Haflingers: *n* = 51, Belgian Draft horses: *n* = 47, Thoroughbreds: *n* = 95, Quarter Horses: *n* = 86), 287 horses for which WGS data were publicly available, and WGS data contributed by the McCue laboratory (*n* = 389). The deletion was only identified in 11 non-Friesian individuals in the heterozygous state (1.15%) (Table 5).

**Table 5:** Number of Horses from Additional Breeds Identified with the 16 kb Deletion on ECA13 Based on Evaluation of 955 Samples

Breed	Horses
Native Mongolian Chakouyi Horse	1
Mangalarga Marchador Horse	1
Sorraia	1
Lipizzaner	4
Unknown Breed	4
Total	11

### Functional investigation

**Fig. 6:**
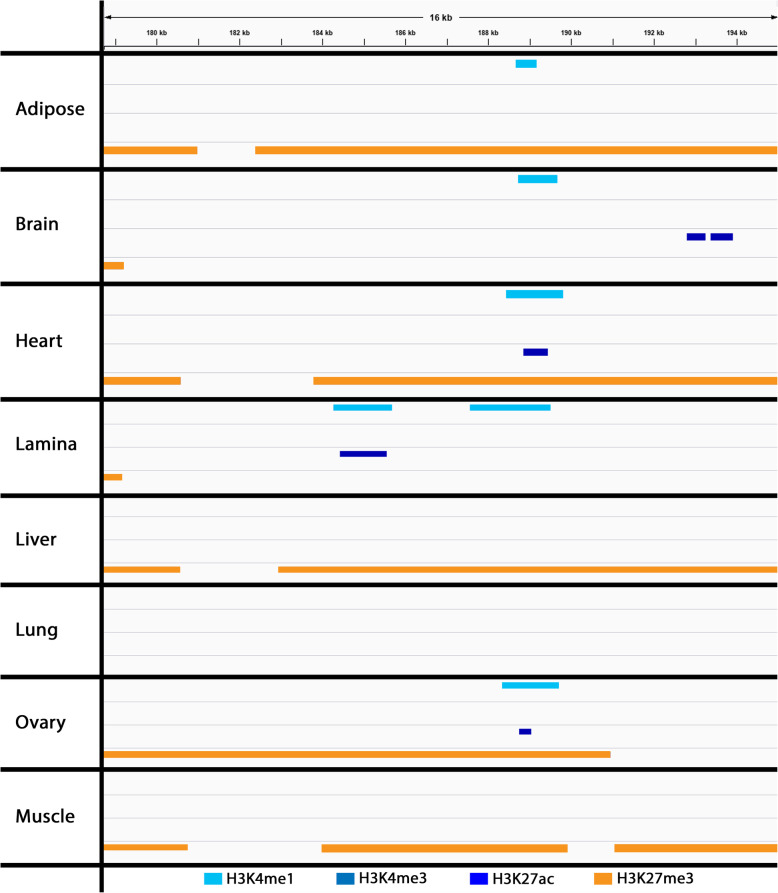
Visualization of Histone Mark Functional Annotation Data in the ECA13 Deletion. BED files of ChIP-Seq histone marks (H3K4me1, H3K4me3, H3K27ac, and H3K27me3) were visually assessed using the Integrative Genomics Viewer (IGV). The diversity of histone marks among these tissues supports the presence of tissue-specific regulatory elements in the ECA13 deletion. Active marks are represented in varying shades of blue and repressive marks are shown in orange

As this locus on ECA13 contains no annotated protein-coding genes and no functional data currently exists for eyelid tissue or Meibomian glands, the equine data collected for the Functional Annotation of Animal Genomes (FAANG) project, including RNA-seq [26, 27] and ChIP-seq data [28] from two clinically healthy Thoroughbred mares, were evaluated to develop additional hypotheses on the functional mechanisms of this variant. To investigate if there was tissue-specific variation in gene expression in clinically normal horses, the three genes near the deletion (*FAM20C*, *PRKAR1B* and *PDGFA*) were assessed in the RNA-seq data. The three genes were expressed in all eight tissues (adipose, brain, heart, lamina, liver, lung, ovary, muscle), which did not help to further elucidate the putative impact of the deletion on gene function. However, in assessing the deletion in the publicly available equine ChIP-Seq data, tissue-specific histone modifications were identified in seven of the eight tissues for which data are available (Fig. 6) [29]. The only tissue with no detected peaks, or genomic locations with significant enrichment for histone interactions, was lung. Interestingly, lamina has evidence of an active enhancer at this locus, with tissue-specific peaks identified for H3K4me1 and H3K27ac [29]; conversely, heart and ovary show evidence of repression in this region indicated by H3K27me3 peaks along with H3K4me1and H3K27ac peaks (Fig. 6) [29]. These findings indicate that there are tissue-specific differences in histone modifications at this locus that could be impacted by the deletion.

## Discussion

A pedigree analysis of Friesian horses affected with distichiasis identified an average inbreeding coefficient of 14.1% in the cohort under investigation, which is higher than that reported for other breeds with closed studbooks (Thoroughbreds = 12.5% and Standardbreds = 8.88%) [30, 31]. In support of this, Schurink et al. 2019 [16] found that the Friesian horse was the most inbred and had the smallest effective population size of the nine breeds they studied based on an analysis of the inbreeding coefficients, the length of ROHs, and measures of linkage disequilibrium. They reported an even higher average inbreeding coefficient 25.5% in the Friesian population they investigated [16]. This high level of inbreeding may explain why Friesians have several reported recessive genetic disorders and supports investigating distichiasis as a recessive trait [17–24].

GWAS and haplotype analyses identified three potential regions of interest (ECA5, ECA12 and ECA13) associated with distichiasis, and additional testing further supported the region on ECA13 (*p* = 1.6 × 10^− 5^). Given that the associations on ECA5 and ECA12 could not be replicated, we concluded these associations were false positives and therefore did not investigate them further.

Interrogation of the associated ECA13 locus using high throughput short-read WGS data did not identify any variants within the coding sequence of the three positional candidate genes. SnpEff identified 32 variants from the associated haplotype, but none were perfectly concordant with the phenotype. The identified 16 kb deletion (ECA13:g.178714_195130del) was also not perfectly concordant with disease but was the most concordant with disease phenotype (cases: *n* = 19 and controls: *n* = 75, *p* = 4.8 × 10^− 13^).

Eighteen out of 19 cases were homozygous for the 16 kb deletion; however, seven out of 75 controls were also homozygous for the deletion. While the controls on average were older than the cases in this study (average age of cases was 8.2 while that of the controls was 12.4 years), how age impacts disease is unknown. It is possible that these seven controls may develop aberrant lashes at some point later in life. It is equally possible that aberrant lashes were in the telogen phase of the hair cycle and thus were not detectable when these seven controls were examined on a single occasion. Reexamination of these seven controls was attempted, but was not possible as the horses were either deceased or not accessible. Alternatively, it is possible that this mutation is incompletely penetrant. This is suspected to be the case for inherited distichiasis in dogs [15]. Horses homozygous for the deletion but not showing signs of disease may have protective genetic variants and/or some environmental trigger maybe impacting disease status. Performing a longitudinal study, with multiple ocular examinations over time to evaluate different phases of the hair cycle in horses genotyped for the ECA13:g.178714_195130del deletion will help to further evaluate the plausibility of incomplete penetrance. Additionally, performing ocular exams and genotyping for the ECA13 deletion on sires and dams of the horses homozygous for the deletion should aid in segregation analysis needed to further evaluate the recessive mode of inheritance proposed and enable a precise estimate of penetrance.

A single horse with distichia was not homozygous for the ROH identified as a part of the GWAS analysis and was heterozygous for the deletion. This case had an atypical presentation relative to the other cases, with only a single unilateral aberrant lash, while the other cases had multiple lashes and/or obvious corneal lesions. This horse was also not available for reexamination.

The estimated allele frequency of ECA13:g.178714_195130del (32.3%) is higher than the allele frequency identified in a subset of this sample for the other recessive conditions reported in this breed (7.5 and 6.2% for hydrocephalus and dwarfism, respectively). This could be because hydrocephalus has fatal consequences and because dwarfism is readily identified and selected against. Additionally, both hydrocephalus and dwarfism are present at birth, thus these cases are identified prior to breeding. While distichiasis can be a significant source of discomfort and a vision-threatening problem in some individuals, some cases may not have obvious clinical signs. Therefore, horses with distichiasis may be bred if they are asymptomatic, have mild clinical signs, or if they have not yet developed the disease, thus propagating the disorder in the Friesian population. It is also possible that this deletion impacts regulation of a gene associated with a favorable phenotype in the breed, and thus is undergoing positive selection.

A study by Conant et al. 2011 [32] reported that Friesians and Haflingers are more closely related than other Iberian breeds, and Schurink et al. 2019 [16] determined that Belgian Draft horses are also closely related to Friesians. Therefore, a sample of phenotyped Haflingers and Belgian Draft horses were evaluated to detect the presence and frequency of this mutation as neither of these closely related breeds are reported to have distichiasis. Quarter Horses and Thoroughbreds were also investigated as more distantly related breeds. The deletion was not detected in any of the 279 horses from these four breeds. However, in evaluating 287 genomes available in the Sequence Read Archive (SRA) and an additional 389 provided by the McCue laboratory comprising of 54 breeds, only 11 horses of diverse breeds not shown to be related to the Friesian breed were identified to have the deletion (Table 5). This suggests that the deletion is not a Friesian-specific mutation, but rather an old mutation that predates the formation of the Friesian breed. Evaluating the frequency of this mutation and its presence in horses that have undergone ocular phenotyping is warranted in the other breeds in which the mutation was identified.

According to the EquCab3.0 reference genome [26], the identified deletion lies in the intergenic region between *FAM20C* and *PDGFA*, which are both included in the associated haplotype identified on ECA13. Because of this, these two genes and the next closest gene, *PRAKR1B*, were considered as positional candidates. Based on known function, *PDGFA* was considered most likely to be involved in distichiasis. The *PDGF* family acts as a paracrine growth factor that mediates epithelial-mesenchymal interactions in various tissue types. As such, *PDGFA* has been shown to play a role in the formation of submandibular salivary glands in mice [33]. In cell culture of submandibular salivary glands, increased levels of *PDGFA* caused an increase in branching and epithelial proliferation [33]. More work is needed to elucidate if *PDGFA* is expressed in normal Meibomian glands and if there is differential expression in distichia-affected glands that leads to the expression of a hair bearing function.

Analysis of the equine FAANG RNA-seq data from two normal horses showed no tissue-specific variation in gene expression for *FAM20C*, *PDGFA*, and *PRKAR1B*, and thus did not provide any insight into potential putative impact of the deletion on gene function. However, putative regulatory elements were identified that could play a role in distichiasis. This study represents the first utilization of the FAANG histone ChIP-seq data to develop hypotheses related to regulatory regions of the genome and illustrates the importance of these datasets as a reference for future investigations. The presence of an active enhancer at this locus, as indicated by H3K4me1 and H3K27ac in lamina, along with evidence of repression in five other tissues with H3K27me3 marks in the deleted region, support this site as a tissue-specific regulatory region. Of the tissues assessed, lamina is the most similar to the ocular lid margin as both tissues have an extensive extracellular matrix. The presence of an active enhancer in lamina suggests that these regulatory elements could be important to Meibomian gland function. We, therefore, hypothesize that an enhancer located in the deleted region plays a role in regulating the expression of *PDGFA* and that deletion of this enhancer site could modify gene expression. In turn, it might cause the Meibomian glands to form a hair-bearing structure as opposed to its normal secretory function, leading to distichiasis. To test these hypotheses, eyelid tissue, including the Meibomian glands, from affected and unaffected horses needs to be investigated using RNA-seq and other functional annotation methods.

The discovery of a novel variant associated with distichiasis will enable genetic testing allowing for marker assisted selection, which provides the opportunity to lower the incidence of the disorder in the Friesian population. It will also help in the identification of horses likely to be affected by distichiasis, thus allowing horse owners to screen horses for the disorder and potentially provide intervention prior to the development of clinical signs and irreversible corneal damage leading to better welfare for these horses.

## Conclusions

This study successfully identified genomic regions associated with distichiasis, and further analysis identified a 16 kb deletion (ECA13:g.178714_195130del) that was associated with the disease phenotype. Given its association and its location in relation to poised and active enhancers, this variant warrants further investigation as causal for distichiasis. Further exploration of the functional consequences of this deletion may help to explain the underlying etiology of distichiasis and penetrance in Friesian horses, humans, dogs and other species.

## Methods

### Samples and DNA extraction

Ninety-nine privately owned, registered Friesian horses were phenotyped for inclusion in this study and remained in their owners’ care for the duration of the study. A complete ocular examination of the adnexa (adjoining surfaces), anterior and posterior segments of both eyes was performed by a diplomate of the European or American College of Veterinary Ophthalmologists. Horses with clinical signs consistent with disease, or with a history of aberrant cilia, were included as cases (*n* = 24, ages from 2 to 16, Fig. 1b). DNA was not available from five of these cases. Unaffected horses had no medical history of aberrant cilia and no aberrant cilia detected on examination (*n* = 75, ages from 1 to 24, Fig. 1a). In the replication sample set, horses with other ocular pathologies were excluded as additional controls. Whole blood and/or mane or tail hair with follicles were collected and genomic DNA was extracted as described in Bellone et al. 2017 [34].

### Pedigree analysis

To investigate possible modes of inheritance for distichiasis, a pedigree analysis was conducted using 8-generation pedigrees from 21 cases and 48 controls. Pedigree information was obtained from the Royal Friesian Horse Studbook (Koninklijke Friesch Paarden Stamboek:KFPS) database [35]. Pedigrees were compiled, visualized and analyzed using Pedigraph [36]. Inbreeding coefficients were calculated for cases and controls and compared using a Mann-Whitney U test [37].

### Genome wide association study

A genome wide association study of 14 cases and 38 controls was performed to identify loci of interest for distichiasis. Genotyping was performed by GeneSeek (Neogen Genomics, Lincoln, NE) using the Axiom 670 k Equine Genotyping array [38]. Analysis and visualization were performed using the Golden Helix SNP and Variation Suite (SVS) [39]. Prior to analysis, the data were remapped to the EquCab3.0 reference genome, reducing the number of SNPs from 670,796 to 636,999 SNPs [40]. Quality control consisted of the exclusion of samples with call rates ≤0.95 and SNPs with call rates ≤0.95 or minor allele frequencies of < 0.05. Based on these criteria, all 52 samples and 299,013 SNPs remained for analysis. The effective number of independent tests (60,027) was calculated using the genetic type 1 error calculator (GEC) with default settings [41]. This number was used to apply a modified Bonferroni significance level (*p* = 8.33 × 10^− 7^). To identify loci of interest, data were analyzed using a chi-squared analysis for basic allelic association. An additive Efficient Mixed-Model Association eXpedited test (EMMAX, F-test) was also performed to correct for genomic inflation [42]. A visual inspection of the haplotype from the genome-wide associated regions was performed and a chi-squared test for independence comparing the frequency of the identified haplotype in cases and controls was completed.

### hapQTL

To refine the GWAS associated regions, a haplotype analysis was performed using hapQTL under default conditions [25]. The analysis was completed on the SNPs from the GWAS that remained after quality control. A BF value of 0.0001 was used as the significance threshold [25].

### Replicating GWAS associations

Thirty-two genome-wide significant SNPs on ECA5, ECA12 and ECA13 were further evaluated to confirm associations identified in the GWAS and haplotype analyses and to replicate findings in additional horses (replication sample set included 5 additional cases and 37 additional controls). One SNP (ECA5:g.39863319A > G) was genotyped using a PCR-RFLP analysis (Table S2) as previously described in Bellone et al. 2017 [34] and the enzyme *BstUI* (New England Biolab Inc., Ipswich, MA, USA) was used to determine genotype based on product size [43]. The remaining SNPs were genotyped using Agena MassARRAY spectrometry, which enables the genotyping of multiple variants simultaneously (Table S3) [44]. Primers were designed using Typer4.0 for MassARRAY [44] and the iPLEX parameter with high multiplexing was utilized with a modification to the primer-dimer potential to 0.8. The default parameters were used for the remaining settings [44]. A Fisher’s exact basic allelic association in SVS was used to compare the allele frequencies between cases and controls for all replication testing.

### Whole genome sequencing

Whole genome sequencing data (Illumina NovaSeq, average 29X coverage with 150 bp PE reads) from 3 Friesians affected with distichiasis and 2 unaffected Friesians were analyzed to identify variants within the ECA13 associated haplotype for concordance with distichiasis. Reads were pre-processed and trimmed using HTSstream [45] with default parameters for all applications except CutTrim, which was used to remove reads shorter than 50 bp. The FASTQ files were aligned to EquCab3.0 using the Burrows-Wheeler Aligner (BWA) [46]. Variants were called using the default parameters in both FreeBayes and SAMtools mpileup [47, 48]. Variants identified by both callers were investigated further. Variant annotation was performed using SnpEff [49].

Because *FOXC2* contains the only known variants to cause distichiasis in humans, the coding region of horse *FOXC2* (ENSECAG00000000843.2) was also assessed using the WGS data [13, 14]. This gene maps to ECA3:g.34103177–34,104,685 in EquCab3.0. The coding regions of the genes present in the 371 kb associated haplotype region on ECA13 plus 1 Mb flanking both the 5′ and 3′ sides of the haplotype were also assessed. Variants were prioritized for further analysis based on: (1) perfect concordance with phenotype in the identified ROH in the samples used for the WGS analysis; and (2) SnpEff predicted functional effect of high, moderate or low regardless of concordance with phenotype in the WGS sample set. These prioritized variants were genotyped in a larger cohort of phenotyped Friesian horses using Agena MassARRAY spectrometry or allele specific PCR (Table S1). Novel variants were archived in the European Variation Archive under accession number PRJEB34362. Primer design was performed as described above, with a modification to the primer-dimer potential to 0.7 [44]. Quality control for these data consisted of the exclusion of samples with call rates ≤0.95, variants with call rates ≤0.98 or with minor allele frequencies of < 0.05. Based on these criteria, all 42 samples and 7 variants remained for analysis. A basic allelic Fisher’s exact test was completed on this data using SVS.

IGV was utilized to assess alignment and read quality and identify potential structural variants [50]. LUMPY [51] was used to confirm visually identified structural variants.

Sanger sequencing of two cases and two controls was performed to identify the boundaries of the ECA13 deletion. Primers and PCR conditions are described in Table S4. The addition of 1 μL BSA was needed to amplify the 3′ end of the deletion. The sequences were compared to the EquCab3.0 reference genome using the NCBI BLAST tool to determine the precise 5′ and 3′ boundaries of the deletion [52]. The deletion was also genotyped in a larger cohort of phenotyped Friesian horses (*n* = 90) using a three-primer allele specific PCR assay (two primers flanking the deletion and one internal primer, Table S4). The amplicons were analyzed on an ABI 3730 Genetic Analyzer and visualized on STRand [53]. A chi-square test for independence was performed to compare the prevalence of the deletion between the cases and the controls. Once the discovery of the deletion was made by our team, a DNA diagnostic test was developed and it is now commercially available at the UC Davis Veterinary Genetics Laboratory. This commercial assay was utilized to genotype the deletion in a random sample of Friesian horses from the Bellone laboratory (*n* = 201). Additional tests available at the UC Davis Veterinary Genetics Laboratory were used to genotype a subset of the Friesian samples (*n* = 73) for the hydrocephalus and dwarfism mutations. DNA samples from other horse breeds, banked in the Bellone laboratory, namely Haflingers (*n* = 51), Belgian Draft horses (*n* = 47), Thoroughbreds (*n* = 95) and Quarter Horses (*n* = 86) were also genotyped for the ECA13 deletion as described above. Of those, all Haflingers and Belgian Draft horses underwent ocular exams and were determined to be unaffected by distichiasis. Thoroughbreds and Quarter Horses were not phenotyped for distichiasis. Mapped equine paired-end WGS BAM files from the SRA (*n* = 192) were assessed for the deletion using LUMPY [51]. Genome STRiP [54] was utilized to evaluate an additional 95 unmapped paired-end equine WGS BAM files from the SRA, as well as 389 horse genomes banked in the McCue laboratory.

### Functional investigation

As the deletion identified is located in an intergenic region, computational analyses were performed to assess the potential regulatory role of this region as the cause of distichiasis. The annotated locations of *FAM20C*, *PDGFA* and *PRKAR1B* from the FAANG RNA-seq data were visualized using IGV [28, 29]. Publicly available equine FAANG ChIP-Seq data (H3K4me1, H3K4me, H3K27ac, and H3K27me3) from eight tissues (adipose, brain, heart, lamina, liver, lung, ovary, skeletal muscle) were assessed to investigate potential regulatory regions [30]. Visualization of regulatory peaks was performed using IGV.

## Supplementary Information

**Additional file 1: Table S1.** WGS Variants Replicated and Validated Using Agena MassArray Spectrophotometry. Novel variants logged in the European Variant Archive (project PRJEB34362). **Table S2**. Primers and PCR Conditions for Amplification of ECA5:g.39863319A > G (AX-103237539). **Table S3**. GWAS SNPs Validated Using Agena MassARRAY Spectrophotometry. **Table S4**. Primers and PCR Conditions for Genotyping and Sequencing ECA13:g.178714-195130del.

## Data Availability

A portion of the datasets generated and/or analyzed during the current study are not publicly available as they are still being investigated for another related project but are available from the corresponding author on reasonable request. The remaining datasets generated and/or analyzed during the current study are available in the European Variation Archive (PRJEB34362: https://www.ebi.ac.uk/ena/browser/view/PRJEB34362, and PRJEB36380: https://www.ebi.ac.uk/ena/browser/view/PRJEB36380).

## Abbreviations

BAM: Binary alignment file
BED: Browser extensible data
BCSL: Bilateral corneal stromal loss
BF: Bayes Factor
ChIP-Seq: Chromatin immunoprecipitation sequencing
ECA: *Equus caballus* chromosome
EMMAX: Efficient Mixed-Model Association eXpedited
FAANG: Functional Annotation of Animal Genomes
GWAS: Genome-wide association study
IGV: Integrative Genomics Viewer
LD: Linkage disequilibrium
PCR-RFLP: Polymerase chain reaction- restriction fragment length polymorphism
ROH: Run of homozygosity
SNP: Single nucleotide polymorphism
SRA: Sequence Read Archive
SVS: SNP and Variation Suite
WGS: Whole genome sequencing
