# Supplementary material for: Whole genome sequencing identified a 16 kilobase deletion on ECA13 associated with distichiasis in Friesian horses
Source: BMC Genomics. 2020 Nov 30;21:848. doi: 10.1186/s12864-020-07265-8 (PMC7706231; doi:10.1186/s12864-020-07265-8)
Supplement: Supplementary file 1 — Additional file 1: Table S1. WGS Variants Replicated and Validated Using Agena MassArray Spectrophotometry. Novel variants logged in the European Variant Archive (project PRJEB34362). Table S2. Primers and PCR Conditions for Amplification of ECA5:g.39863319A > G (AX-103237539). Table S3. GWAS SNPs Validated Using Agena MassARRAY Spectrophotometry. Table S4. Primers and PCR Conditions for Genotyping and Sequencing ECA13:g.178714-195130del. [file 12864_2020_7265_MOESM1_ESM.docx]

**Table S1. WGS Variants Replicated and Validated Using Agena MassArray Spectrophotometry.** Novel variants logged in the European Variant Archive (project PRJEB34362).

| **Name** | **Name** | **Name** | **Name** |
| --- | --- | --- | --- |
| ECA13:g.117852G>A  rs1147761652 | ECA13:g.333227C>T  rs396327454* | ECA13:g.565673AG>ACTAG** | ECA13:g.1334883A>C** |
| ECA13:g.124255A>T  rs1149145282* | ECA13:g.438278AA>CC** | ECA13:g.565872G>A  rs782910380** | ECA13:g.1334890C>T** |
| ECA13:g.125711T>C  rs1137818600 | ECA13:g.440085A>G  rs396062387** | ECA13:g.710940T>C rs395586130 | ECA13:g.1335562C>T  rs1140888961** |
| ECA13:g.127995G>A rs1147801946 | ECA13:g.452679G>A** | ECA13:g.711850G>A  rs1135913968** | ECA13:g.1336141A>G  rs396496589** |
| ECA13:g.134862C>G rs1144199873 | ECA13:g.453135C>T* | ECA13:g.781123T>C  rs394628691** | ECA13:g.1336288T>C  rs395194524* |
| ECA13:g.138340G>A rs1135947419 | ECA13:g.472842G>A  rs395826931** | ECA13:g.781417T>C rs395847330** | ECA13:g.1336299C>T** |
| ECA13:g.158596G>A | ECA13:g.472865G>A rs394643577** | ECA13:g.1200743CCAC>ACAT rs1143730718** | ECA13:g.1336762C>T* |
| ECA13:g.302205G>A rs396901711** | ECA13:g.495551A>G rs396693160** | ECA13:g.1201099C>G** | ECA13:g.1337062T>C** |

*Variant failed to genotype.

** Variant did not pass quality control due to a minor allele frequency of <0.05.

**Table S2**. **Primers and PCR Conditions for Amplification of ECA5:g.39863319A>G (AX-103237539).**

| **Region** | **Forward Primer (5’-3’)** | **Reverse Primer (5’-3’)** | **Annealing Temp. °C** | **Number of Cycles** |
| --- | --- | --- | --- | --- |
| ECA5 SNP | TTCCCAGTCACGACGTTG  CAAGGCTCCCAAGATCTGAC | TGCCGGATAAGTTACCAGAGA | 60 | 38 |

**Table S3. GWAS SNPs Validated Using Agena MassARRAY Spectrophotometry.**

| **Affymetrix Number** | **Name** | **Affymetrix Number** | **Name** |
| --- | --- | --- | --- |
| AX-104460242 | ECA13:g.13611A>G | AX-103461747 | ECA13:g.201534G>A |
| AX-103773680 | ECA13:g.15928A>G | AX-103446586 | ECA13:g.206996G>A |
| AX-103233306 | ECA13:g.32112G>T | AX-103676668 | ECA13:g.230097C>T |
| AX-103150334 | ECA13:g.42897A>G | AX-104606028 | ECA13:g.316346C>T |
| AX-103121605 | ECA13:g.121325G>A | AX-104370759 | ECA13:g.330462G>A |
| AX-103334838 | ECA13:g.134040A>G | AX-105010310 | ECA13:g.340918C>T |
| AX-103816621 | ECA13:g.142416C>T | AX-104470326 | ECA13:g.343850A>C |
| AX-104975847 | ECA13:g.153435C>T | AX-103493706 | ECA13:g.384663T>C |
| AX-103758787 | ECA13:g.186975G>A | AX-104539485 | ECA13:g.425443C>T |
| AX-105011287 | ECA13:g.189306C>T | AX-103452967 | ECA13:g.122064G>A |
| AX-104139867 | ECA13:g.190216T>C | AX-103682283 | ECA13:g.122243C>T |
| AX-104370825 | ECA13:g.195278T>C | AX-103710679 | ECA5:g.39962594G>A |
| AX-102978266 | ECA13:g.198581G>C | AX-103235594 | ECA12:g.3270900T>C |
| AX-103543678 | ECA13:g.200759T>C | AX-104785965 | ECA12:g.3271275C>A |

**Table S4**. **Primers and PCR Conditions for Genotyping and Sequencing ECA13:g.178714-195130del.**

| **Region** | **Forward Primer (5’-3’)** | **Reverse Primer (5’-3’)** | **Annealing Temp. °C** | **Number of Cycles** |
| --- | --- | --- | --- | --- |
| 5’ end of the deletion | TGGCATCTGTCTGTCCATCT^*^ | CTCCTGGAACCTCCTGGAAC | 58 | 37 |
| 3’ end of the deletion | AGCCTAGTCCTGCCCCCTA | ACCCGCAAGCTGTTTCTTT | 58 | 37 |
| Internal | TGGCATCTGTCTGTCCATCT* | ACCCGCAAGCTGTTTCTTT | 58 | 37 |

*FAM labeled primer for genotyping assay. Unlabeled primer used in sequencing reactions.
